# Sanborn on Medical Education

**Published:** 1853-08

**Authors:** J. E. Sanborn

**Affiliations:** Professor of Chemistry and Materia Medica


### IOWA MEDICAL JOURNAL.

VOL. I. KEOKUK, IOWA, AUGUST, 1853. NO. 1.

### ORIGINAL COMMUNICATIONS.

### *Importance of a Sound Medical Education.*

From an ADDRESS to the last Graduating Class of the Iowa Medical College,

BY J. E. SANBORN, M. D.,
Professor of Chemistry and Materia Medica.

What more precious amulet can be wreathed about the hearts of you, who to-day close the term usually devoted to professional pupilage, than the adoption of those great principles of duty and integrity, which will of themselves be a sufficient safe-guard in every trying scene which may await you. How can we better express our solicitude for your welfare, than in the devotion of this last hour to the enunciation of such sentiments as we deem most important for a successful pilgrimage in life. * * * * *

Few periods of one's career are of more vital importance, more pregnant with the interests of the vast and dim future, than that hour when he passes from the quiet and peaceful contemplation of great ideas, to their active and vivid embodiment in practical life. Thus far, it has been the period of studious thought—of the quiet laying up of important truths ; but henceforth, these truths are to be sown broad-cast, and germinate into actions. I can conceive of hardly one thing, centering in itself more emotions of interest, than the carving out into actual, tangible life, of great, long cherished principles. It is such a delicate balancing of hope and expectation, with just enough of fear to give to the incidents an almost breathless interest, that it seems to concentrate the hoarded thoughts of years, into the throbbing emotions of an hour. With what intensity of interest, does the inventor, who has for years brooded over an obstinate problem of mechanics, gaze upon the final result—so thrillingly successful—when the ponderous sinews of iron enginery, perform with accurate obedience, the duty he so long ago predicted.

And those who have felt it, will recall how infinitely above this is the keener pleasure of the physician, when he sees the great principles of his science, producing their legitimate and beautiful results, in the actual life around him. When, after long days of darkness and doubt, lest disease shall sullenly refuse to restore to us its victim, the glad sunshine of returning health breaks in, once more, upon the heart, and the strengthening pulse and the brightening eye are a gladdening tribute to the successful application of correct principles.

This future, then, of earnest action is before you. While you pause upon its threshold, let us review together some of the esssential requisites of a good physician. That qualification which I place first as of predominant importance is, of course, *sound medical education.* Who would trust himself in the midst of the shallows, and quicksands, and tortuous currents of our mighty river to the care—if we dare call it such—of one who knew nothing of the winding and invisible pathways, but would steer with ignorant recklessness upon its treacherous surface. You know, too well, the treachery of disease—how it sometimes lures the unsuspecting, almost as if designing to deceive; and you know that none but a skillful pilot is fitted to guide the trembling bark of life over those darksome and perilous waters.

To speak of the necessity of a thorough medical education, would only be to utter one of the most common-place convictions of every day life—for we expect our hod carriers to be endowed with an accurate idea of their duties, and an ability to perform them faithfully. Upon no one point does the community more rigidly insist, than upon the faithful execution of voluntarily assumed duties; and upon no one thing does the community more thoroughly and properly visit its indignation, than upon a failure to perform pretended offices. Mankind, the world over, condemn and ridicule pretension, and are generally not wanting in ability to detect it. I say it, not to frighten, but to admonish.

You need not, I am sure, be harangued upon the importance of a sound medical education; and yet, some brief considerations may not be without a use, in re-assuring us of its necessity. If we are to construct an edifice, as a permanent residence, with what care do we select an architect; we demand the proofs of his capability; we examine his integrity, intelligence and skill, and we inspect with studious eye, the structures he has already raised. But how much more important are these vital structures in which we daily live and move and have our being, and infinitely do they surpass in value, the inanimate structures which we raise, with our own hands ; and of what intelligence and skill should he be possessed, in whose care is to rest the preservation of these our animate residences.

Such accuracy of skill is not always, however, demanded in the medical architect. The community, doubtless, are somewhat at fault in this respect. To the shame of the community, rather than of the medical profession, be it said that quacks are sustained as physicians, who, as architects, would not be responsible contractors for the successful construction of a hen-coop. I have placed this sin at the door of society. I know it is very common, in our easy language, to speak of such parasites as a "disgrace to the profession," and many persons, by some indefinite process of logic, contrive to lay the blame at the hands of medical men, as a class. I cannot see how. The medical profession did not educate them—nobody has done that—it does not encourage or support them ; and the blame of their existence rests with those who *do*,—the community, at large.

Let, however, the existence of such external sources of annoyance be a subject of philosophic consolation to the true physician. They subserve, as do all snch beings, a useful purpose. They are supported by an undiscriminating portion of the people, with this good effect : that thereby the difference between the false and the true may only be the more palpably visible. Be not annoyed by their presence. Let such perversions of the great and noble art of our common zeal, but stimulate you to the highest attainments, that you may, both to your own heart, and in the view of a discerning public, set yourself as far from them as possible.

Your first effort, and aim, then, is to be thoroughly prepared for the proper and successful discharge of your professional duties. This, of course, is the corner stone of success ; without it. you will in vain attempt to rear any enduring superstructure ; but, upon it, you may easily erect a lasting reputation. The possession, then, of a thorough and entire preparation for your future labors, is *indispensable* ; for, as much as the human system, with its wonderful mechanism and divine excellence, is above an engine, of wood or iron, as much as the life of a man is above the life of a machine, *so* much should the sphere of a physician's labor and fitness, be above that of an ordinary artisan. But, *why* is this ? why should you aim at the most complete preparation? Not, because, by such a course you can most easily obtain your needed food and clothing; this you can do, in the honest and healthful profession of the wood-sawyer. Not even, because, by such a course, your name will be of more regard, and your position more respectable. But, because honesty, integrity and duty demand it. By following such a course, *alone*, can you meet the ends of an elevated and high minded nature; for, if you satisfy yourself with merely common-place attainments, such as are barely sufficient to answer your daily need, without exposure to open ridicule and contempt, you will be false to the world, to whom you offer your services, and what is more, false to yourself.

A physician, by offering his services to the community, virtually says to the world, that he is prepared to assume the duties and perform the responsible offices usually expected of an educated medical man. Such is his language to the world; but how do his *actions* speak? If he be negligently incompetent to perform his duties, his professional life is a daily repeated falsehood. His *position* assures you that he is adequate to the discharge of his duties—the real amount of his attainments, on the other hand, palpably gives the lie to the language of his station! and what a position is this! How intensely unsatisfactory, not to say dishonorable! The great charm of a sphere of active and successful usefulness in life, results from a sort of conscious integrity—a conviction that one is prompted by honorable motives, and is fully able to perform all that he assumes to himself, or all that his position demands. Such a conviction should ever be your constant companion. It is the guardian of that elevated self respect, without which, life has little of honor or worth. There is no skulking in the heart of a thoroughly honorable man. He has nothing to be ashamed of. There are no depths of ignorance deep within his bosom, which he is solicitous to cover up and conceal from the world. And yet, such an one is not presumptuous or arrogant—far from it; these are qualities of the ignorant pretender, who seeks, by clouds of learned dust, to cover his ignorance, and who boasts, in advance, in order that no presuming countryman may dare to question his learning. The arduous responsibilities of a medical life are enough, of themselves—they do not need to be augmented by a harrassing consciousness of imperfect preparation—by the constant fear of incompetent discharge of duty.

We do not expect that any one, will, in the brief period of one life-time, become familiar with all that is known of our science; but it is most assuredly within the province of every one, to learn enough to enable him to act intelligently, confidently and successfully. A sensitive spirit can have no more sure and direct source of permanent misery, than to be crowded with the duties of a profession, for which he were conscious he has but an incomplete preparation. While, on the other hand, few spheres of life are capable of affording so much and so elevated a pleasure, as a consciousness of a proper fitness for that station, whose office it is to perform the blessed alchemy of changing misery, sickness and gloom, to happiness, health and sunshine.

A sense of duty, then, of high minded integrity, and even a regard for one's own happiness, all stimulate to the attainment of an honorable preparation for the career of a successful and good physician. But, other motives still, concentrate upon the same point. The community are oftentimes, ill informed respecting the character and attainments of the medical profession ; and, as a consequence, are sometimes ill disposed towards those who represent it. These prejudices generally occur, I may say, from erroneous impressions. The opinion respecting a whole class has been based upon the unworthiness or incompetence of a few. There is need, therefore, of enlightenment, in this respect. Erroneous opinions which have been originated by an unworthy physician, and fostered by ignorance, must be eradicated by a more liberal education. The medical profession, as a class, must be represented by more worthy specimens. Every young physician who passes forth from the halls of medical instruction, goes as an apostle of medical science. He is, in a certain sense, the representative of the whole class of medical men ; to the sphere in which he moves, he is a type of the true physician ; and from him, hundreds may make up their ideas of all which is becoming and needed in one of that class.

See to it, then, that you bear well the standard of your and our common reputation ; have a care, that your deportment, and especially your intellectual attainments, may reflect honor upon your vast brotherhood. Fail not, then, each in his individual place and capacity, to vindicate your class from the erroneous imputation of ignorance and incompetence. Show to those whom you influence, the intelligence and enlightened liberality which should ever grace the charac- of the true physician.

Even however—to recur—if we presume to flatter ourselves that we begin our life of medical practice with a fair intellectual capital, we must not dare to suppose that it will long answer our purpose. It is indeed, a poor style of business transaction, in which we constantly draw upon our resources, but never replenish them. No error is at once so ridiculous, and disastrous, as to suppose, for an instant, that one has *ever* completed his education. Imagine, for a moment, that, by some strange law of our nature, it would be impossible for us to gain any knowledge, after a certain age ; that, after the line of say thirty years, the human mind could take no steps forwards. What zeal would stimulate our studies up to that charmed period, and with what tearful lamentations should we mourn the day, that cut us off from all further growth in mental stature ! But yet, how many seem, by their indifference, to consider that fatal day already past ; how many move on, with the portals of the mind all closed, as if the seal of leaden lethargy had already been stamped upon it. But a kind Providence has so ordained it, that the years, which plow their furrows upon the brow, and limit the strength of the arm, and check the speed of the foot, shall set no barrier to the outgoings of the triumphant intellect.

Life, then, is a school ; and constant study, ceaseless acquisition of knowledge, the only means by which we can honorably acquit ourselves during the period of our life-long pupilage. Especially is this true of the physician, when the constant advance of science renders him hardly less than criminal, if he does not zealously bring all the resources of modern improvements, to bear upon the great object which he has placed before himself. * * * * *

How quiet and noiseless is the sphere of the proper student ! During the weeks and months that have passed, we have been constantly pursuing the even tenor of our way. We have come and gone, upon the silent errands of our daily toil, and few have ever taken note of our movements. Our passings to and fro have been of no importance amid the hurried jostlings of our busy streets, and yet, of what significance may have been the quiet teachings of the past season ! The important truths, the great vital principles which have been the theme of our instructions, shall have henceforth a wider field—a more extended application. Hereafter, they shall live in more hearts than before, and scores of hands shall now minister to suffering and necessity.

It is a beautiful reflection that the guiding principles of professional life, which may be enunciated in a few weeks, admit of such endless application and are of such eternal truth, that they will live, both in mens' hearts and in mens' practice, when countless ages shall have rolled away. In such a view of the case, how momentous the position of a medical teacher ; that he may so teach, that none shall ever suffer by his errors.—He occupies, indeed, an office of high trust. If we have taught error, the first patient who seeks your counsels, may pay for our blunders the forfeit of his life! The tares may grow together with the wheat, till both attain their full growth, in the ripeness of intellectnal maturity.

If we have, at any time, failed to enunciate with precision, the truth, not only yourselves, but the whole community, would be the victims of our imperfect teaching. Not of such a cast, however, have been our labors. We have earnestly endeavored, and we may say it without impropriety, so to expound the principles of our science, that your course may be a pathway of light, rather than of darkness. But, if the position of the professional teacher be one of eminent importance, and respectability, hardly less so is that of the medical pupil: so to learn, that he shall correctly apply the principles laid down. For, the pupil of to-day will be the teacher of to-morrow. Each physician shall instruct and educate, for good or for bad, the hundreds who look to him for professional guidance, and trustingly confide in his medical instruction. Though his voice may never be heard in the halls of popular instruction, yet his character and influence shall be a speaking lesson to hundreds, perhaps thousands, around him. * *

To-day, the relation of teacher and pupil is sundered. To-day are loosed the chords, that have bound you to the incidents of a season; henceforth, a wider sphere of action opens before you. Pass forth to it, with trusting hope, and a manly reliance. Regard not the world as cold, heartless and unfeeling; it is so only to those who think it such; and they who thus esteem it, are they who make it thus. In the breast of each one is the light which illumines the world around him. Act always up to the full measure of your intelligence and duty. Sow, in youth, the seeds of such actions as shall germinate into great results, and shall honor and protect your old age with their kindly and benevolent foliage. So live—

"That on the tablet of life's setting sun,

Shall be engraved, as with a diamond pen,

'A LIFE OF DUTY DONE.'"

